# Supplementary material for: Reduced Oxidative Stress and Enhanced FGF21 Formation in Livers of Endurance-Exercised Rats with Diet-Induced NASH
Source: Nutrients. 2019 Nov 8;11(11):2709. doi: 10.3390/nu11112709 (PMC6893460; doi:10.3390/nu11112709)
Supplement: Supplementary file 1 [file nutrients-11-02709-s001.pdf]

Reduced oxidative stress and enhanced FGF21 formation in livers of endurance-exercised rats with diet-induced NASH

Janin Henkel, Katja Buchheim-Dieckow, José P. Castro, Thomas Laeger, Kristina Wardelmann, André Kleinridders, Korinna Jöhrens, Gerhard P. Püschel

Supplementary Tables & Figures

Supplementary Table S1: Sequences of the forward and reverse primers used for RT-qPCR. The primer sequences were derived on the basis of the given accession numbers and were checked for specificity by BLAST search.

Gene	Forward (5'-3')	Reverse (5'-3')	Acc. No.
Eef2	CTGAAGCCTATCCAGAGGACC	AGACTCCTCGATGATGCACTG	NM_017245.2
F4/80 (Adgre1)	CTCTTCTGGGGCTTCAGTGG	GCAGACTGAGTTAGGACCACA	NM_001007557
Fasn	GTGATAGCCGGTATGTCCGG	GGCGTCGAACTTGGACAGAT	NM_017332.1
Fgf21	CAGATGACGACCAGGACAC	AAGTGAGGCGATCCATAGAG	NM_130752.1
Glucokinase	GCCGTGCCTGTGAAAGCGTGTC	CCACCCGTAGCAGCAGAATAGGTC	NM_001270849.1
Glut1	TCTGCCGTGCTTATGGGTTT	CCGAACACCTGGGCAATAAG	NM_138827
Hprt1	TCCCAGCGTCGTGATTAGC	CCAGCAGGTCAGCAAAGAAC	NM_012583.2
IL-1β	TCGACAGTGAGGAGAATGACC	GAAGGTGCTTGGGTCCTCAT	NM_031512.2
Irs2	GACGAGTACTTCGCCGTAGC	TGTTCGCAATTGAGCTTCAC	NM_001168633.1
mt-Nd1	CTATTCGGAGCCCTACGAGC	AATAGGGCGAATGGTCCTGC	NC_001665.2
Pepck	GCAGAGCATAAGGGCAAGGTCA	CCAGCACGCGGGAGTTCT	NM_198780.3
Srsf4	AGGGACGCAAAAACGAAGGA	CGGGAATGTCTGCTTCGAGA	NM_001108685.2
Ucp3	CTTGGCTAGACTCACAGGCAG	GTGCGCACCATAGTCAGGAT	NM_013167.2
β-actin (Actb)	GCGAGTACAACCTTCTTGCAG	GTTAAGCTTTGCCAGTGTCCG	NC_005111.3

Supplementary Table S2: Additional parameters measured in plasma, liver and soleus muscle. Rats were subjected to the different treatment groups described in the legend to Figure 1. Values are mean ± SEM. Statistics: Statistics: Two-way-ANOVA with Tukey's post hoc test for multiple comparisons; #: significant versus sedentary standard-diet group, §: significant versus exercised standard-diet group, * significant versus sedentary NASH-diet-group. $p < 0.05$, (*1): $p = 0.053$, (§2): $p = 0.051$.
Abbreviations: ASAT: aspartate aminotransferase, ALAT: alanin aminotransferase, FGF21: fibroblast growth factor 21, Glut1: glucose transporter 1, IRS: insulin receptor substrate, Pepck: phosphoenolpyruvate carboxykinase.

	**standard-diet sed**	**standard-diet run**	**NASH-diet sed**	**NASH-diet run**
Plasma analysis				
ASAT [U/L]	71.57 ± 3.27	81.13 ± 5.88	123.13 ± 11.17 #	149.43 ± 18.63 # §
ALAT [U/L]	43.43 ± 4.70	53.38 ± 5.01	69.50 ± 7.16	53.88 ± 8.09
Triglycerides [mmol/L]	0.64 ± 0.08	0.37 ± 0.04 #	0.27 ± 0.01 #	0.23 ± 0.01 #
Cholesterol [mmol/L]	1.53 ± 0.08	1.67 ± 0.12	2.77 ± 0.19 #	2.44 ± 0.12 # §
Free fatty acids [mmol/L]	0.28 ± 0.01	0.32 ± 0.02	0.24 ± 0.02 #	0.19 ± 0.02 # §
Liver analysis				
Fatty acid synthase (Fasn) mRNA	1.29 ± 0.32	0.39 ± 0.06 #	0.19 ± 0.04 #	0.14 ± 0.03 #
Fatty acid synthase (Fasn) protein	1.00 ± 0.16	0.79 ± 0.13	0.48 ± 0.09 #	0.31 ± 0.08 # (§2)
IRS2 mRNA	1.07 ± 0.12	0.78 ± 0.09	0.87 ± 0.08	0.28 ± 0.04 # § *
IRS2 protein	1.0 ± 0.19	0.77 ± 0.11	0.70 ± 0.12	0.34 ± 0.04 #
Phospho-$Ser_{307,612/632}$-IRS1 protein	1.00 ± 0.11	0.80 ± 0.09	0.65 ± 0.06	0.55 ± 0.14 #
Glucokinase mRNA	1.46 ± 0.47	0.77 ± 0.17	2.05 ± 0.44	0.69 ± 0.13 (*1)
Pepck mRNA	1.05 ± 0.07	1.22 ± 0.08	0.41 ± 0.05 #	0.38 ± 0.03 # §
Soleus muscle analysis				
Phospho-$Ser_{307,612/632}$-IRS1 protein	1.00 ± 0.13	1.17 ± 0.14	1.98 ± 0.41 #	1.40 ± 0.20
FGF21 mRNA	1.04 ± 0.09	1.14 ± 0.11	1.32 ± 0.15	1.27 ± 0.10
Glut1 mRNA	1.02 ± 0.07	0.84 ± 0.07	1.06 ± 0.16	0.66 ± 0.06 *

Supplementary Table S3: Results of the comparable animal study with Sprague Dawley rats. Rats were subjected to the different treatment groups described in the legend to Figure 1 with 4 animals per group. Values are mean ± SEM. Statistics: Statistics: Two-way-ANOVA with Tukey's post hoc test for multiple comparisons and Kruskal-Wallis test with Dunn's post hoc test for multiple comparisons (non-parametric data: NASH activity score); #: significant versus sedentary standard-diet group, §: significant versus exercised standard-diet group, p < 0.05.
Abbreviations: ASAT: aspartate aminotransferase, AUC: area under the glucose curve in the i.p. glucose tolerance test, IL-1β: Interleukin-1β.

	**standard-diet sed**	**standard-diet run**	**NASH-diet sed**	**NASH-diet run**
Body weight gain: AUC [g x 9 weeks]	4331.6 ± 150.1	4215.9 ± 4.3	4629.3 ± 211.1	4426.1 ± 80.9
Plasma analysis				
AUC [(mg/dL x 120 min) / 1000]	17.39 ± 1.09	17.73 ± 0.78	22.98 ± 2.30	18.74 ± 1.13
ASAT [U/L]	82.25 ± 5.09	73.25 ± 3.45	102.75 ± 6.14	128.00 ± 25.99
Triglycerides [mmol/L]	0.67 ± 0.22	0.45 ± 0.06	0.30 ± 0,02	0.28 ± 0.01
Cholesterol [mmol/L]	2.08 ± 0.19	1.72 ± 0.23	2.33 ± 0.02	2.12 ± 0.33
Liver analysis				
NASH activity score	0.50 ± 0.29	0.75 ± 0.25	6.50 ± 0.65 #	6.00 ± 0.71 # §
Diagnosis no NAFLD	4	4	0	0
NAFLD	0	0	2	2
NASH	0	0	2	2
Triglycerides [mg/mg protein]	0.42 ± 0.08	0.41 ± 0.07	1.31 ± 0.19 #	1.16 ± 0.14 # §
Cholesterol [mg/mg protein]	0.10 ± 0.01	0.09 ± 0.01	2.08 ± 0.45 #	1.55 ± 0.27 # §
F4/80 mRNA	1.03 ± 0.13	1.23 ± 0.14	3.82 ± 0.83 #	2.57 ± 0.24
IL-1β mRNA	1.03 ± 0.12	1.05 ± 0.10	4.53 ± 0.97 #	3.14 ± 0.26

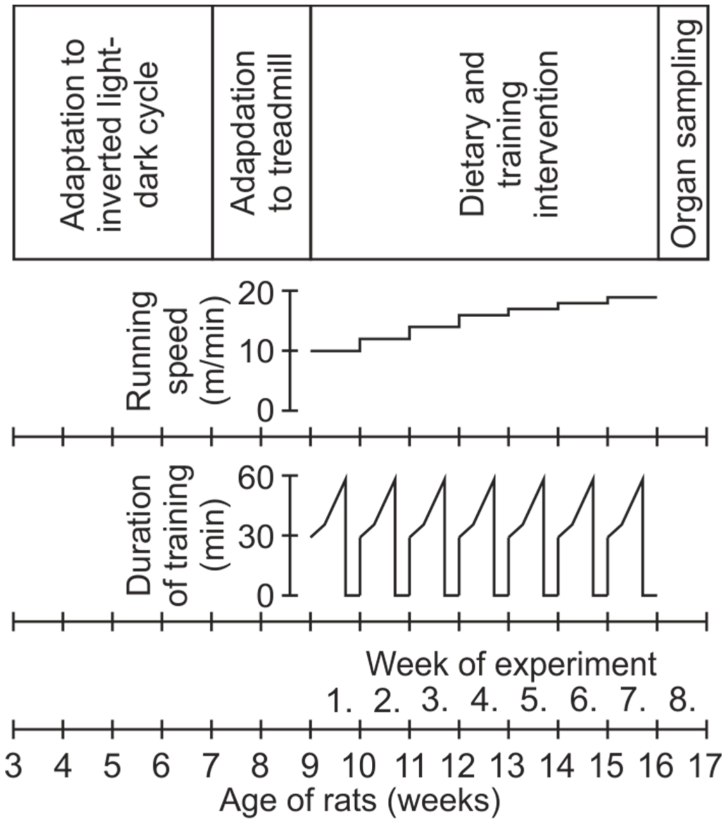

Supplementary Figure S1: Schematic representation of the intervention. At the age of three weeks, weaned rats were adapted to an inverted light/dark cycle (light from 8:30 p.m. to 8:30 a.m.), to allow training in the dark phase (8:30 a.m. to 8:30 p.m.), which corresponds to the active phase of the animals. After 7 weeks, all rats were adapted to the treadmill for two weeks, first allowing the rats to explore the immobile treadmill and then increasing speed and duration daily from 8 min/min for 5 min to a maximum of 15 m/min for 30 min. After two weeks of adaptation, rats were randomly assigned to one of the four intervention protocols: Sedentary standard-diet, sedentary NASH-diet, running standard-diet or running NASH-diet. Rats were trained for 5 days per week and rested for 2 days. The training was started at a running speed of 10 m/min for 30 min. During the week the training duration was stepwise increased to 60 min. In the subsequent week, speed was increased to 12 m/min and the duration of the training was again increased from 30 to 60 min throughout the week. This scheme of increasing load and intensity was continued over the 7 weeks of intervention resulting in a maximum performance of 60 min running at 19 m/min. Rats were rested for 48 h prior to organ sampling. No training was performed on the day of glucose tolerance test.

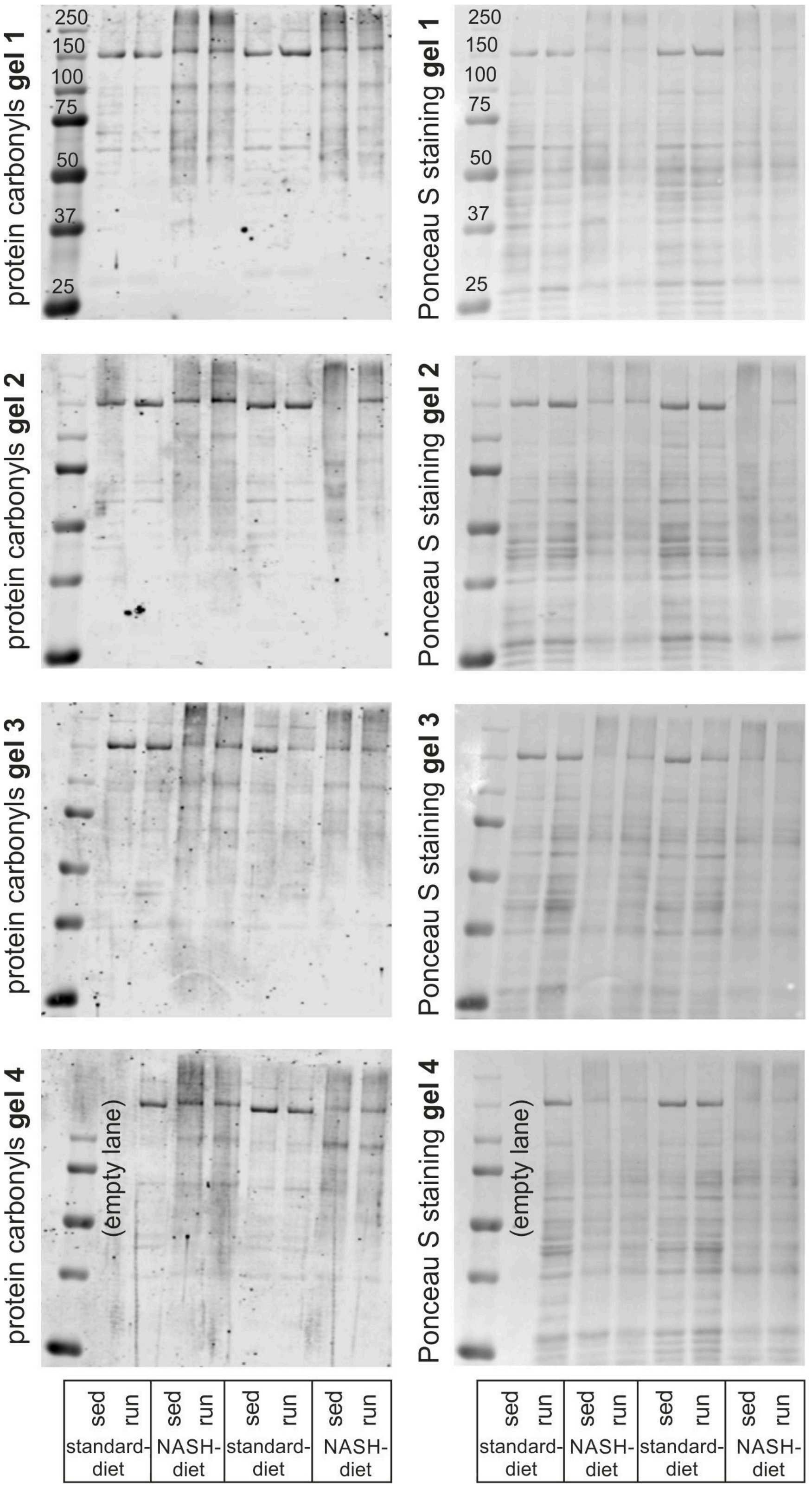

Supplementary Figure S2: Detection of protein carbonyls by oxyblot analysis in liver homogenates. For quantification dense intensity of each lane from 250 to 50 kDa in oxyblots and Ponceau S staining were determined.

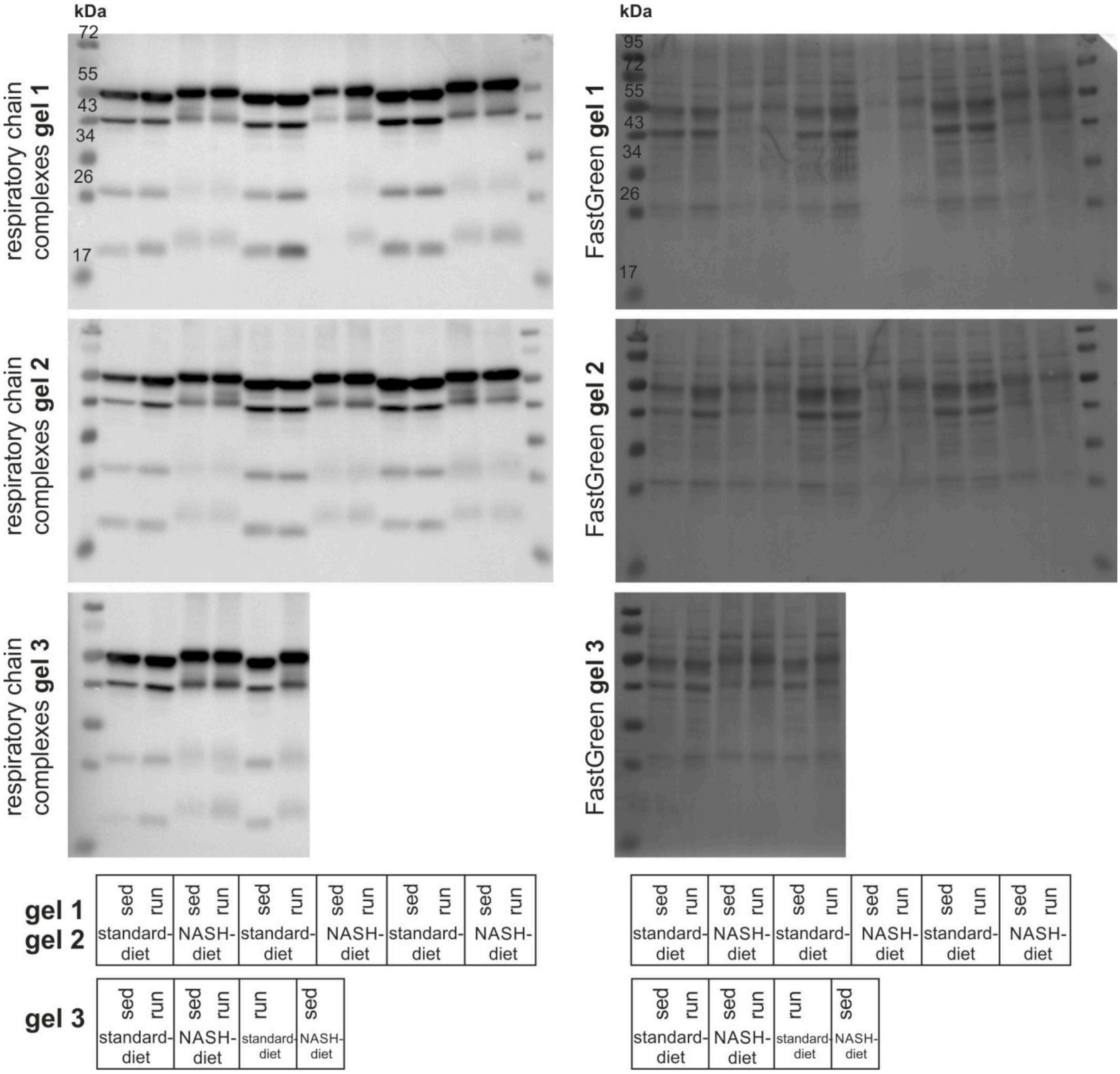

	standard-diet sed	standard-diet run	NASH-diet sed	NASH-diet run
**complex I expression [arbitrary units]**	1.00 ± 0.19	1.02 ± 0.11	0.59 ± 0.05	0.85 ± 0.08 *
**complex II expression [arbitrary units]**	1.00 ± 0.12	1.15 ± 0.10	0.59 ± 0.06 #	0.57 ± 0.03 # §
**Sum all complexes [arbitrary units]**	1.00 ± 0.13	1.07 ± 0.08	0.75 ± 0.06	0.83 ± 0.04

Supplementary Figure S3: Detection of the complexes of the respiratory chain in liver homogenates. For quantification dense intensity of complex I (~ 20 kDa), complex II (~30 kDa), complex III (~40 kDa) and complex V (~55 kDa) as well as FastGreen-staining (density of each lane from ~ 20 - 75 kDa) were determined. Statistics: Two-way-ANOVA with Tukey's post hoc test for multiple comparisons; #: significant versus sedentary standard-diet group, §: significant versus exercised standard-diet group, * significant versus sedentary NASH-diet-group (with Student's T-Test). $p < 0.05$.

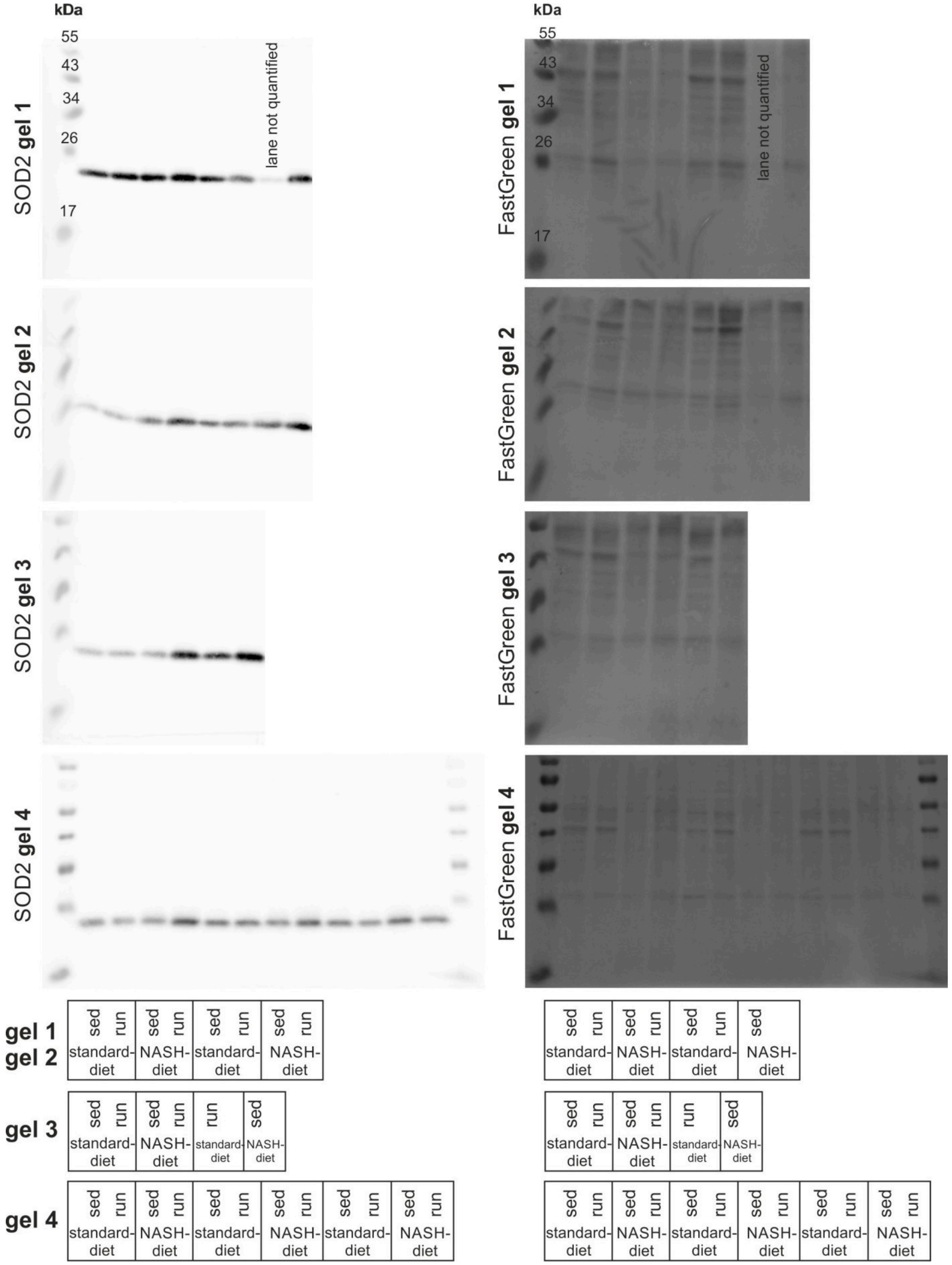

Supplementary Figure S4: Detection of superoxide dismutase 2 (SOD2) in liver homogenates. For quantification dense intensity of SOD2 (22 kDa) and FastGreen-staining (density of each lane from ~ 20 - 55 kDa) were determined.

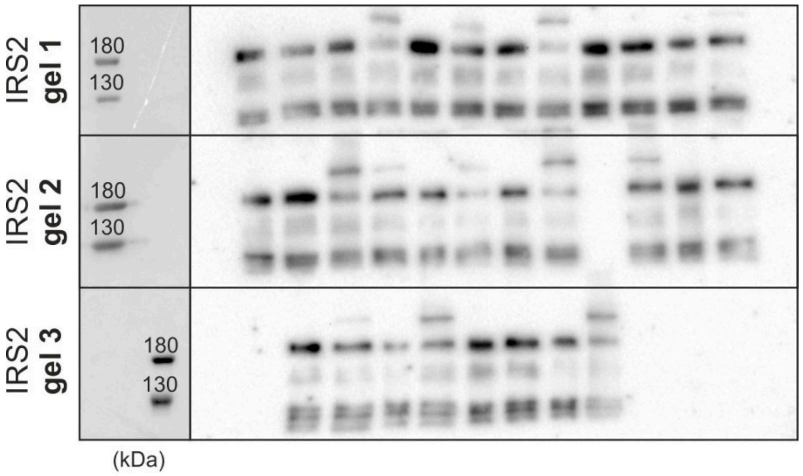

	sample order **gel 1**, **gel 2**	sample order **gel 3**
lane 1	standard-diet sed	
lane 2	NASH-diet sed	standard-diet sed
lane 3	standard-diet run	NASH-diet sed
lane 4	NASH-diet run	standard-diet run
lane 5	standard-diet sed	NASH-diet run
lane 6	NASH-diet sed	standard-diet sed
lane 7	standard-diet run	NASH-diet sed
lane 8	NASH-diet run	standard-diet run
lane 9		NASH-diet run
lane 10	NASH-diet sed	
lane 11	standard-diet run	
lane 12	NASH-diet run	

Supplementary Figure S5: Detection of IRS2 protein expression in liver homogenates. SDS-PAGE with 7.5 % acrylamide was performed and gels were cut on a horizontal line (about 100 kDa). The upper parts with range > 100 kDa of 3 gels were blotted on one membrane. After incubation with an anti-IRS2 antibody the visualization of the immune complexes revealed more than one band. This is caused by the shift of the apparent molecular weight of IRS2 by partial or complete phosphorylation of several phosphorylation sites of the protein. Therefore, the dense intensity of all bands in the range between ~180 kDa and ~130 kDa was included for quantification.

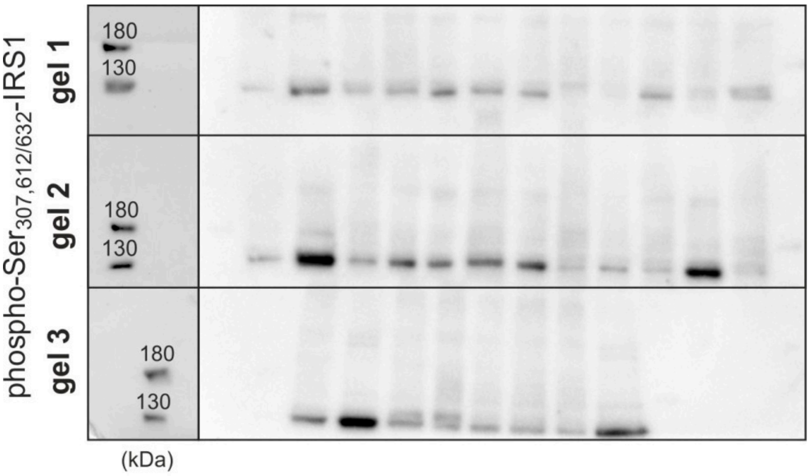

	sample order **gel 1**, **gel 2**	sample order **gel 3**
lane 1	standard-diet sed	
lane 2	NASH-diet sed	standard-diet sed
lane 3	standard-diet run	NASH-diet sed
lane 4	NASH-diet run	standard-diet run
lane 5	standard-diet sed	NASH-diet run
lane 6	NASH-diet sed	standard-diet sed
lane 7	standard-diet run	NASH-diet sed
lane 8	NASH-diet run	standard-diet run
lane 9	standard-diet sed	NASH-diet run
lane 10	NASH-diet sed	
lane 11	standard-diet run	
lane 12	NASH-diet run	

Supplementary Figure S6: Detection of phospho-$Ser_{307,612/632}$-IRS1 protein expression in soleus muscle homogenates. SDS-PAGE with 7.5 % acrylamide was performed and gels were cut on a horizontal line (about 100 kDa). The upper parts with range > 100 kDa of 3 gels were blotted on one membrane. After incubation with a mixture of phospho-specific anti-Ser_{307}-IRS1 and anti-$Ser_{612/632}$-IRS1 antibodies, the visualization of the immune complexes revealed one band with a molecular weight of ~130 kDa, which is ~50 kDa below the calculated molecular weight of IRS1. This is due to a phosphorylation-dependent shift in the apparent molecular weight. In addition, several bands with apparent molecular weights between 180 and 130 were visible. These bands most likely correspond to partially phosphorylated IRS1 molecules. Therefore, the dense-intensity of the entire molecular weight range between 180 kDa and 130 kDa was determined to quantify phospho-Ser-IRS1.
